# Ultrasound and clinicopathological features of papillary thyroid carcinomas with BRAF and TERT promoter mutations

**DOI:** 10.18632/oncotarget.22430

**Published:** 2017-11-14

**Authors:** Soo Yeon Hahn, Tae Hyuk Kim, Chang Seok Ki, Sun Wook Kim, Soohyun Ahn, Jung Hee Shin, Jae Hoon Chung

**Affiliations:** ^1^ Department of Radiology and Center for Imaging Science, Samsung Medical Center, Sungkyunkwan University School of Medicine, Seoul, Korea; ^2^ Division of Endocrinology and Metabolism, Department of Medicine, Samsung Medical Center, Sungkyunkwan University School of Medicine, Seoul, Korea; ^3^ Department of Laboratory Medicine, Samsung Medical Center, Sungkyunkwan University School of Medicine, Seoul, Korea; ^4^ Biostatistics and Clinical Epidemiology Center, Research Institute for Future Medicine, Samsung Medical Center, Sungkyunkwan University School of Medicine, Seoul, Korea

**Keywords:** papillary thyroid carcinoma, BRAF mutation, TERT promoter mutation, ultrasound, prognosis

## Abstract

This study is to investigate if any relationship exists between the telomerase reverse transcriptase (TERT) promoter or proto-oncogene BRAF mutation and ultrasound (US) and clinicopathological features of papillary thyroid carcinomas (PTCs). The study included 150 patients with surgically confirmed PTC from October 1994 to December 2004. According to the existence of TERT promoter or BRAF mutations, we categorized patients into three groups (no mutation, BRAF mutation alone, or TERT+BRAF mutations) and analyzed the relationships between TERT promoter or BRAF mutation and US and clinicopathological features. The rate of recurrence or death according to mutation analysis was estimated. There were 35 (23.3%) cases with no mutation, 104 (69.3%) with BRAF mutation alone, and 11 (7.3%) with TERT+BRAF mutations. As the number of genetic mutations increased from no mutation to BRAF mutation alone to both BRAF and TERT mutations, the proportions of hypoechogenicity, non-parallel orientation, spiculated/microlobulated margin, microcalcifications, and high suspicion category increased. PTCs with TERT+BRAF mutations recurred more frequently than other groups (odd ratio = 17.921 and 31.468). The intervals to recurrence and overall survival were significantly shorter in the TERT+BRAF mutation group than in the other groups (*P*s <.0001). PTCs with no mutation, with BRAF mutation alone, and with both TERT and BRAF mutations linearly increase in the probability of displaying malignant US features. In PTCs, the coexistence of BRAF with TERT mutations is more strongly correlated with recurrence and mortality than BRAF mutation alone.

## INTRODUCTION

Papillary thyroid carcinoma (PTC) is the most common type of thyroid cancer, accounting for 70–90% of well-differentiated thyroid malignancies [1]. In most cases, PTC generally displays an indolent clinical course and has excellent prognosis despite 15–30% local or regional recurrence [2–4]. However, some PTCs exhibit more aggressive characteristics and may cause mortality. Consequently, various risk stratification methods have been used for the appropriate management of patients with thyroid cancer; however, none are completely accurate.

In recent years, molecular biomarkers have been investigated as adjunct diagnostic markers of thyroid cancer and as predictors of patient prognosis. According to several studies, BRAF mutations are associated with poor prognostic factors, including larger tumor size, older age, male gender, extrathyroidal extension, tumor multifocality, lymph node metastasis, advanced tumor stage, and recurrence [5–9]. However, the prognostic impact of BRAF mutations in patient mortality remains controversial [10, 11]. Therefore, additional prognostic biomarkers to predict aggressive disease are needed.

The human telomerase reverse transcriptase (TERT) gene encodes the catalytic reverse transcriptase subunit of telomerase, and its function is to maintain chromosomal integrity and genome stability [12]. Somatic mutations of the promoter region of this gene, found at -124 and -146 from the start of the translational ATG site, have been reported in various cancers, including thyroid tumors [13–15]. In thyroid cancers, TERT promoter mutations were predominantly found in aggressive disease, such as tall cell variant PTC, widely invasive follicular thyroid carcinoma, poorly differentiated carcinoma, and anaplastic carcinoma [5, 15–17].

Through previous studies, it was clarified that preoperative ultrasound (US) findings are strongly correlated with the clinical behavior and prognosis of thyroid cancers [18–21]. Although Xing et al. recently reported the prognostic value of BRAF and TERT promoter mutations in PTC [22], to our knowledge, there have been no studies that have classified US imaging characteristics of PTCs based on molecular biomarkers including BRAF and TERT promoter mutations. The aim of our study was to investigate if any relationship exists between TERT promoter or BRAF mutation and US and clinicopathological features of PTCs.

## RESULTS

The BRAF mutation was found in 69.3% (104 of 150) of PTCs, whereas the TERT promoter mutation was found in 7.3% (11 of 150) of PTCs. Therefore, there were 35 cases (23.3%) in the no mutation group, 104 (69.3%) in the BRAF mutation alone group, and 11 (7.3%) in the TERT+BRAF mutation group.

As shown in Table 1, patient age at the diagnosis of PTC was significantly older in the TERT+BRAF mutation group than in the no mutation or BRAF mutation alone group (*P* <.0001). Extrathyroidal extension of PTC, surgical margin involvement, lateral lymph node metastasis, advanced TNM stage (stage III/IV disease), recurrence, and death were more common in the TERT+BRAF mutation group than in the BRAF mutation alone group (*P* =.0052, *P* =.0089, *P* = 0.0002, *P* =.0007, *P* <.0001, and *P* =.0001, respectively). Central lymph node metastasis was more frequently observed in the BRAF mutation alone or TERT+BRAF mutation group compared to that in the no mutation group (*P* =.0465). The mean duration of clinical follow up was 149.6 ± 24.6 months (range, 61.4–210.6 months) and the duration of imaging follow up ranged from 60.0 to 224.0 months, with a mean of 143.1 ± 34.5 months. At the time of last follow up, five patients died of thyroid disease: four in the TERT+BRAF mutation group and one in the BRAF mutation alone group. The causes of death were local recurrence (n = 2), lung metastases (n = 2), and brain metastasis (n = 1).

**Table 1:** Relationship between mutation analysis results and clinicopathological characteristics of 150 papillary thyroid carcinomas

Clinicopathological characteristics	No mutation (*n* = 35)	BRAF mutation alone (*n* = 104)	BRAF + TERT mutations (*n* = 11)	Overall *P* value
Age (mean ± SD, years)	39.0 ± 12.3	43.5 ± 12.3	61.3 ± 13.3	< 0.0001^*^
< 45 years	32.7 ± 7.2	34.7 ± 7.8	38.2	
≥ 45	54.6 ± 6.6	54.1 ± 7.0	63.6 ± 9.7	
Sex				0.5716
Women	31 (88.6)	84 (80.8)	9 (81.8)	
Men	4 (11.4)	20 (19.2)	2 (18.2)	
Type of lymph node dissection				0.0209^*^
Central neck dissection	22 (62.9)	78 (75.0)	4 (36.4)	
Both central and lateral neck dissection	13 (37.1)	26 (25.0)	7 (63.6)	
Tumor characteristics at surgery				
Size (mean ± SD, cm)	3.2 ± 1.1	2.9 ± 1.2	3.1 ± 0.9	0.2022
Multiplicity	12 (34.3)	30 (28.9)	3 (27.3)	0.8142
Extrathyroidal extension	18 (51.4)	75 (72.1)	11 (100)	0.0052^*^
Surgical margin involvement	3 (8.6)	10 (9.6)	5 (45.5)	0.0089^*^
Central lymph node metastasis	13 (37.1)	63 (60.6)	5 (45.5)	0.0465^*^
Lateral lymph node metastasis	9 (25.7)	18 (17.3)	8 (72.7)	0.0002^*^
Aggressive subtype	0 (0)	1 (1.0)	0 (0)	1.000
Pathologic TNM stage				0.0007^*^
Early stage (stage I/II)	26 (74.3)	59 (56.7)	1 (9.1)	
Advanced stage (stage III/IV)	9 (25.7)	45 (43.3)	10 (90.9)	
Radioiodine scan				0.7604
None	2 (5.7)	2 (1.9)	0 (0)	
Diagnostic dose	32 (91.4)	99 (95.2)	11 (100)	
Therapeutic dose	1 (2.9)	3 (2.9)	0 (0)	
Follow up period				
Clinical follow up (mean ± SD, months)	153.3 ± 25.1	148.8 ± 23.0	145.7 ± 37.5	0.5570
Imaging follow up (mean ± SD, months)	145.7 ± 31.2	143.3 ± 29.9	144.5 ± 43.1	0.9250
Recurrence	4 (11.4)	13 (12.5)	8 (72.7)	< 0.0001^*^
Death	0 (0)	1 (1.0)	4 (36.4)	0.0001^*^

^*^A *P* value of <.05 was considered statistically significant. SD, standard deviation; TNM, tumor node metastasis.

Extrathyroidal extension was found in 100% of PTCs with TERT+BRAF mutation.

The relationship between mutation analysis results and US imaging characteristics is summarized in Table 2. PTCs with BRAF or TERT+BRAF mutations exhibited hypoechogenicity more frequently than PTCs without any mutation (*P* =.0029). PTCs with TERT+BRAF mutation had more instances of non-parallel orientation and spiculated/microlobulated margin than PTCs with BRAF mutation alone or with no mutation (*P* =.0094 and *P* <.0001, respectively). According to the results of the Cochran-Armitage trend test, as the number of genetic mutations increased, the proportions of hypoechogenicity (*P* =.0066), non-parallel orientation (*P* =.0023), spiculated/microlobulated margin (*P* =.0364), microcalcifications (*P* =.0822), and high suspicion category (Korean Thyroid Imaging Reporting and Data System [K-TIRADS] category 5, *P* =.0670) increased. Meanwhile, as the number of genetic mutations increased, the proportions of isoechogenicity (*P* =.0162) and ill-defined margin (*P* <.0001) decreased. Common US features of PTCs with BRAF+TERT demonstrated solid (91%), hypoechoic (73%), non-parallel orientation (73%), microlobulated margin (91%), and the presence of microcalcifications (91%). Cases with all five malignant features were present in 5 (45.5%) of 11 PTCs with BRAF+TERT, 19 (18.3%) of 104 PTCs with BRAF, and 2 (5.7%) of 35 PTCs with no mutation (*P* =.0090).

**Table 2:** Relationship between mutation analysis results and us imaging characteristics of 150 papillary thyroid carcinomas

US imaging characteristics	No mutation (*n* = 35), *n* (%)	BRAF mutation alone (*n* = 104), *n* (%)	BRAF + TERT mutations (*n* = 11), *n* (%)	Overall *P* value	*P* value for trend
Composition				1.000	
Solid	31 (88.6)	92 (88.4)	10 (90.9)		.8922
Predominantly solid	4 (11.4)	11 (10.6)	1 (9.1)		.8268
Predominantly cystic	0 (0)	1 (1.0)	0 (0)		.7628
Echogenicity				.0029^*^	
Isoechoic	15 (42.9)	16 (15.4)	3 (27.3)		.0162^†^
Hypoechoic	11 (31.4)	69 (66.4)	6 (54.5)		.0066^†^
Markedly hypoechoic	9 (25.7)	19 (18.3)	2 (18.2)		.3985
Shape				.1824	
Oval-to-round	22 (62.9)	74 (71.1)	5 (45.5)		.7834
Irregular	13 (37.1)	30 (28.9)	6 (54.5)		
Orientation				.0094^*^	
Parallel	26 (74.3)	53 (51.0)	3 (27.3)		.0023^†^
Non-parallel	9 (25.7)	51 (49.0)	8 (72.7)		
Margin				<.0001^*^	
Smooth	11 (31.4)	49 (47.1)	1 (9.1)		.9402
Ill-defined	8 (22.9)	1 (1.0)	0 (0)		<.0001^†^
Spiculated/microlobulated	16 (45.7)	54 (51.9)	10 (90.9)		.0364^†^
Calcifications				.1899	
Microcalcification	22 (62.9)	75 (72.1)	10 (90.9)		.0822^†^
Others	13 (37.1)	29 (27.9)	1 (9.1)		
Final K-TIRADS assessment				.0555	
Category 2 (Benign)	1 (2.8)	0 (0)	0 (0)		0.1131
Category 3 (Low suspicion)	0 (0)	0 (0)	0 (0)		
Category 4 (Intermediate suspicion)	17 (48.6)	30 (28.9)	4 (36.4)		0.1168
Category 5 (High suspicion)	17 (48.6)	74 (71.1)	7 (63.6)		0.0670^†^
Doppler US				.2402	
No vascularity	2 (5.7)	14 (13.5)	1 (9.1)		0.4049
Peripheral vascularity	1 (2.8)	1 (1.0)	1 (9.1)		0.5987
Mild central vascularity	6 (17.1)	24 (23.1)	2 (18.2)		0.6747
Prominent central vascularity	9 (25.7)	16 (15.4)	4 (36.4)		0.8887
Not applicable	17 (48.6)	49 (47.1)	3 (27.3)		0.3620

^*^ A *P* value of <.05 was considered statistically significant.

^†^A *P* value for trend is calculated by Cochran-Armitage trend test and considered significant if *P* value <.1.

US, ultrasound; K-TIRADS; the Korean Thyroid Imaging Reporting and Data System.

**Figure 1:**
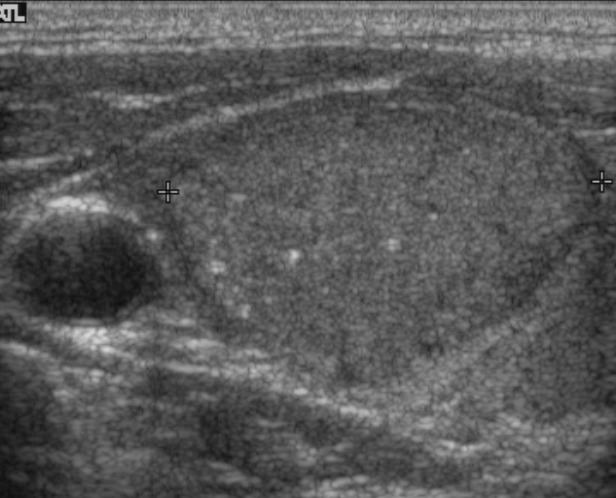

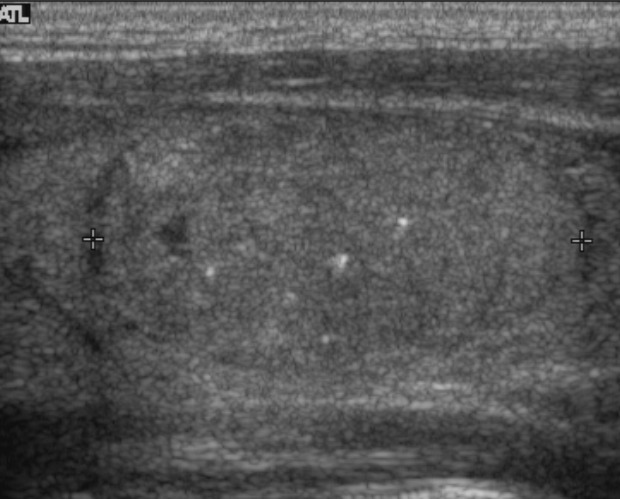
A 44-year-old man with a papillary thyroid carcinoma without any mutation in right thyroid lobe Transverse **(A)** and longitudinal **(B)** ultrasonographic images show a 3.0-cm solid mass with circumscribed margin, oval-to-round shape, isoechogenicity, parallel orientation, and microcalcifications. This mass was classified as K-TIRADS category 4. After surgery, there was no lymph node metastasis and TNM stage was classified as I. There was no recurrence during 10.9 years of follow-up.

**Figure 2:**
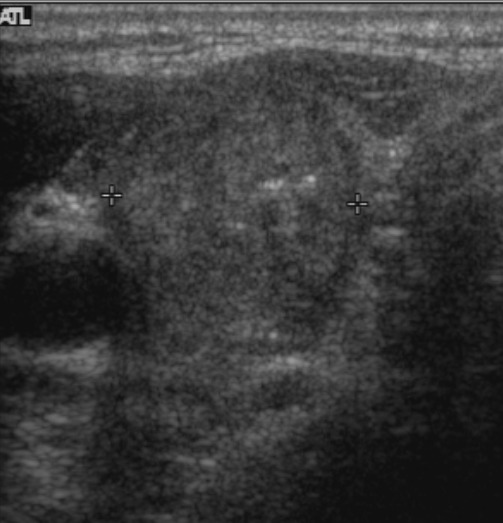

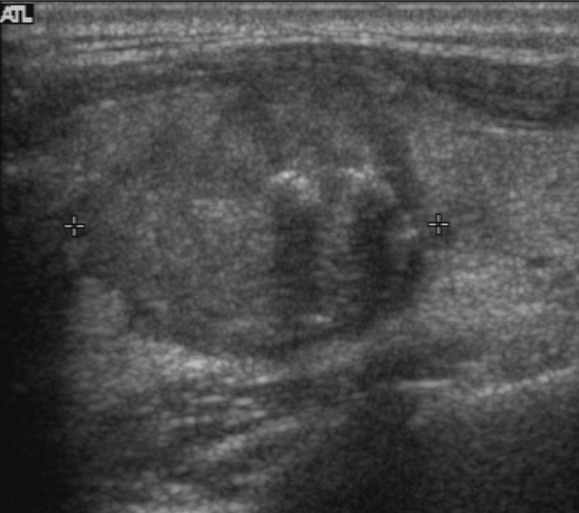
A 52-year-old woman with a papillary thyroid carcinoma with BRAF mutation in right thyroid lobe Transverse **(A)** and longitudinal **(B)** ultrasonographic images show a 2.2-cm solid mass with spiculated margin, irregular shape, hypoechogenicity, non-parallel orientation, and micro- and macrocalcifications. This mass was classified as K-TIRADS category 5. After surgery, central lymph nodes were confirmed as metastases and TNM stage was classified as III. There was no recurrence during 11.4 years of follow-up.

**Figure 3:**
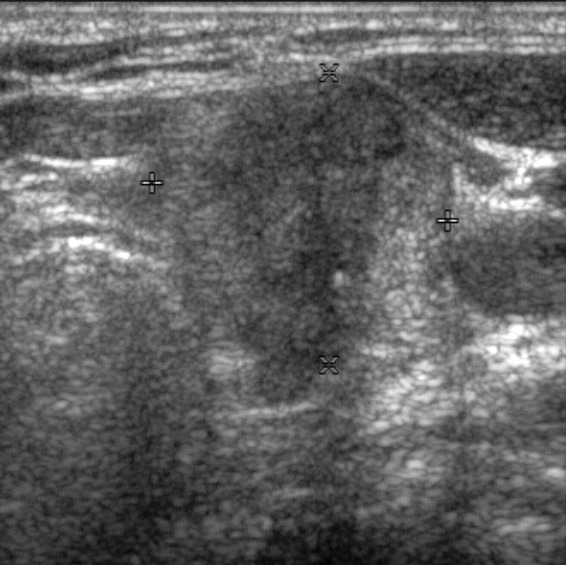

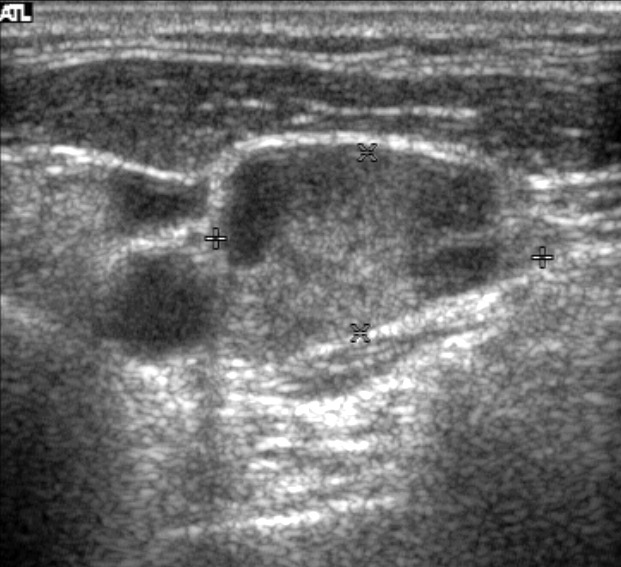
A 56-year-old woman with a papillary thyroid carcinoma with TERT+BRAF mutations in left thyroid lobe Transverse **(A)** ultrasonographic image shows a 3.0-cm solid mass with spiculated margin, irregular shape, hypoechogenicity, non-parallel orientation, and microcalcifications. Transverse **(B)** ultrasonographic image shows an enlarged lymph node with increased cortical echogenicity and cystic changes in left level IV. The main mass was classified as K-TIRADS category 5 and the level IV lymph node was considered metastatic. After surgery, level IV lymph nodes were confirmed as metastases and TNM stage was classified as IV. Bilateral lung metastases and operative bed recurrences were diagnosed 4.4 years after surgery.

Upon multinomial logistic regression analysis (Table 3), PTCs with BRAF mutation alone showed significantly higher frequencies of extrathyroidal extension (odd ratio [OR] = 2.443, 95% confidence interval [CI] = 1.109–5.378), central lymph node metastasis (OR = 3.863, 95% CI = 1.417–10.531), hypoechogenicity (OR = 8.061, 95% CI = 2.994–21.705), and non-parallel orientation (OR = 4.098, 95% CI = 1.497-11.217) than PTCs without any mutation (Figures 1 and 2). PTCs with TERT+BRAF mutation had lateral lymph node metastasis more frequently than PTCs with BRAF mutation alone (OR = 22.557, 95% CI = 2.181–233.294) and demonstrated non-parallel orientation on US more frequently than PTCs without any mutation (OR = 11.282, 95% CI = 1.008–126.270) (Figures 1 and 3). PTCs with TERT+BRAF mutation recurred more frequently than PTCs with BRAF mutation alone and PTCs without any mutation (OR = 17.921, 95% CI = 2.187–146.848, and OR = 31.468, 95% CI = 2.704–366.263, respectively). There was also a significant association between TERT+BRAF mutations and mortality (OR = 51.500, 95% CI = 5.130-516.973). When the age matched comparison was performed, the recurrence and mortality in patients with PTC were also significantly correlated with TERT+BRAF mutations (OR for recurrence, TERT+BRAF mutation vs. no mutation group, 31.125 [95% CI = 2.503–387.022, *P* =.0075]; OR for recurrence, TERT+BRAF mutation vs. BRAF mutation alone group, 20.979 [95% CI = 2.380–184.907, *P* =.0061]; OR for mortality, TERT+BRAF mutation vs. BRAF mutation alone group, 21.355 [95% CI = 1.244–366.514, *P* =.0348]).

**Table 3:** Multinomial logistic regression analysis of 150 papillary thyroid carcinomas

Characteristics	BRAF mutation alone^*^		BRAF + TERT mutations^*^		BRAF + TERT mutations^†^	
	OR (95% CI)	*P* value	OR (95% CI)	*P* value	OR (95% CI)	*P* value
Clinicopathological factors						
Age ≥ 45	2.444 (0.582–10.259)	.2219	5.620 (0.005–6382.362)	.6306	2.299 (0.002–2320.753)	.8135
Extrathyroidal extension	2.443 (1.109–5.378)	.0266^‡^	-	-	-	-
Surgical margin involvement	1.007 (0.203–4.991)	.9933	8.439 (0.640–111.212)	.1050	8.381 (0.920–76.325)	.0593
Central lymph node metastasis	3.863 (1.417–10.531)	.0083^‡^	0.836 (0.084–8.359)	.8785	0.216 (0.026–1.816)	.1584
Lateral lymph node metastasis	0.492 (0.156–1.552)	.2261	11.091 (0.909–135.392)	.0595	22.557 (2.181–233.294)	.0089^‡^
TNM stage III + IV	1.174 (0.280–4.917)	.8267	11.010 (0.009–13315.884)	.5077	9.382 (0.009–10115.858)	.5298
Recurrence	1.756 (0.387–7.961)	.4654	31.468 (2.704–366.263)	.0059^‡^	17.921 (2.187–146.848)	.0072^‡^
Death	-	-	-	-	51.500 (5.130–516.973)	.0008^‡^
US imaging factors						
Hypoechoic	8.061 (2.994–21.705)	<.0001^‡^	2.856 (0.316–25.840)	.3504	0.354 (0.047–2.678)	.3146
Non-parallel orientation	4.098 (1.497–11.217)	.0060^‡^	11.282 (1.008–126.270)	.0492^‡^	2.753 (0.283–26.780)	.3830

^*^Multinomial logistic regression analysis with no mutation group as reference.

^†^Multinomial logistic regression analysis with BRAF mutation alone group as reference.

^‡^A *P* value of <.05 was considered statistically significant.

US, ultrasound; OR, odds ratio; CI, confidence interval.

**Figure 4:**
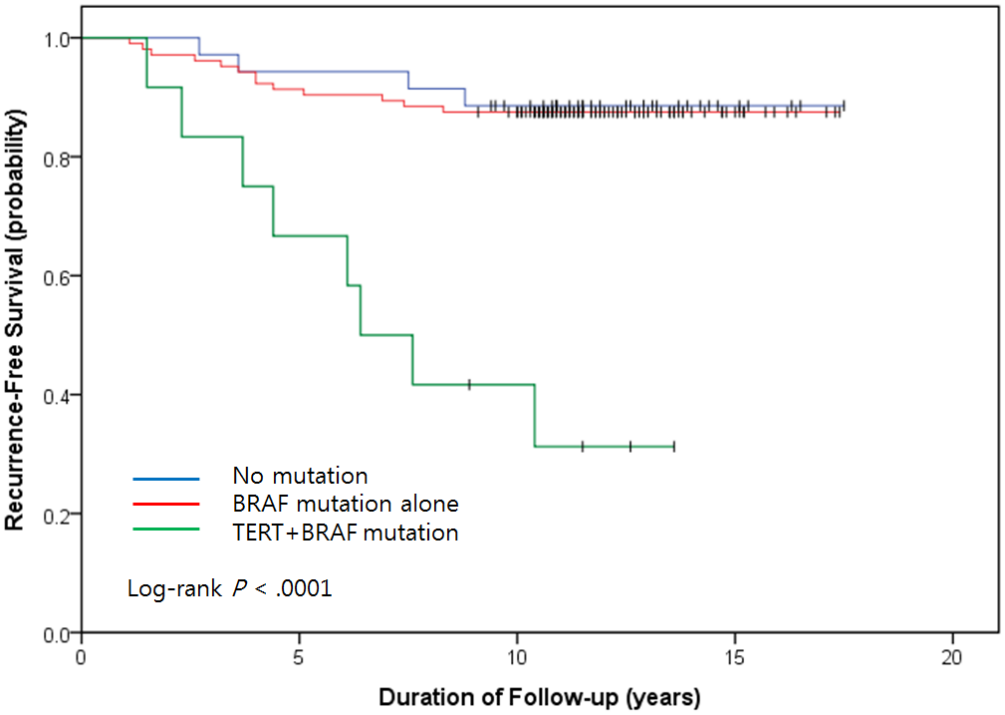

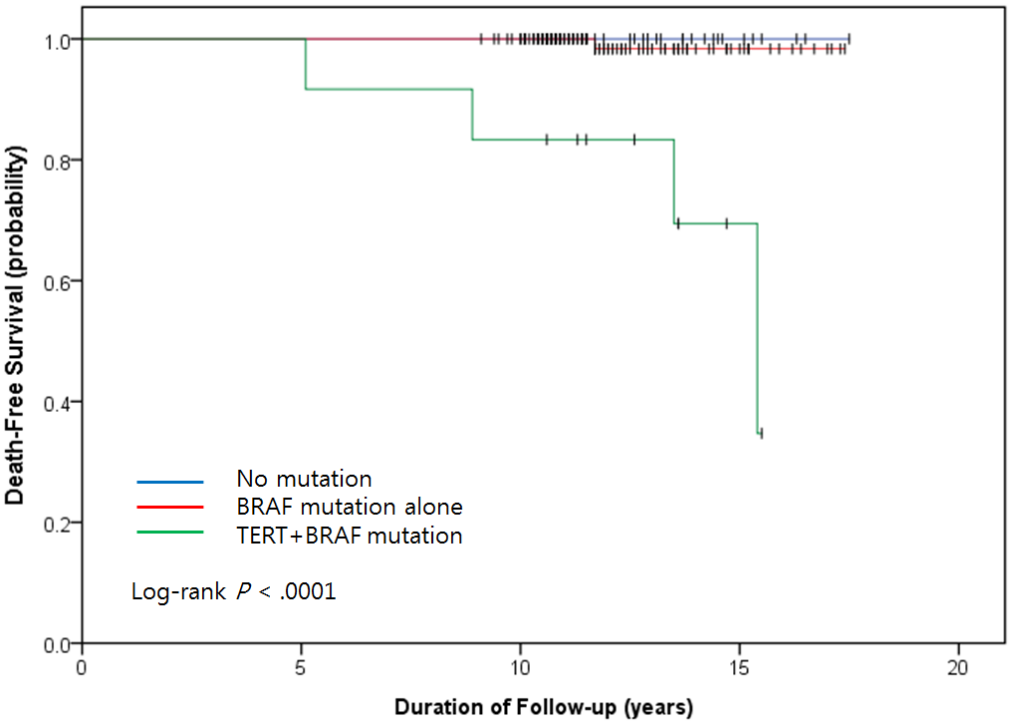
Kaplan-Meier analyses of the three groups on recurrence-free interval and death **(A)** Recurrence-free interval in patients with papillary thyroid carcinoma (PTC). **(B)** Death in patients with PTC. Blue lines represent patients negative for the indicated mutation. Red lines represent patients positive for the BRAF mutation. Green lines represents patients positive for the TERT mutation.

A significantly shorter interval to recurrence was observed for the TERT+BRAF mutation group (median, 76.8 months) relative to the no mutation (median, 140.4 months) and BRAF mutation alone groups (median, 138.1 months) (*P* <.0001; Figure 4A). Patients in the TERT+BRAF mutation group also had a significantly shorter overall survival duration (median, 135.6 months) than patients in the no mutation (median, 153.6 months) or BRAF mutation alone group (median, 153.6 months) (*P* <.0001; Figure 4B).

## DISCUSSION

In this study on BRAF and TERT promoter mutations in PTC, we found a significant association between TERT+BRAF mutations and the development of aggressive clinicopathologic features. We also demonstrated that the TERT+BRAF mutation is a strong predictor of recurrence and mortality of PTC. In our study, the prevalence of TERT promoter mutations was 7.3%. This prevalence was consistent with previous studies, reporting rates of 7.3–25.5% [15, 17, 22–25]. Several previous studies reported an association between BRAF and TERT promoter mutations in PTC [14, 22, 26]. Despite the debate on the effect of coexisting BRAF and TERT promoter mutations in PTC [17, 24], Xing et al. [26] and Jin et al. [27] demonstrated that this feature has important prognostic value and is strongly associated with poor clinicopathologic outcomes in PTC. In our study, compared to the BRAF mutation alone group, TERT+BRAF mutations were associated with lateral lymph node metastasis, recurrence, and mortality in PTC. In addition, the TERT+BRAF mutation group showed a significantly shorter interval to recurrence or death than the BRAF mutation alone or no mutation group. The BRAF mutation alone group was associated with extrathyroidal extension and central lymph node metastasis when compared to PTCs of the no mutation group.

According to previous studies [28–31], US features of malignant thyroid nodules are well established, and include hypoechogenicity, spiculated/microlobulated margin, microcalcifications, and non-parallel orientation. These suspicious US findings are relatively consistent regardless of histologic type or variant of thyroid cancer [18, 32–34]. Moreover, several studies demonstrated that US characteristics of thyroid nodules might be highly reliable for predicting clinical outcomes and appropriate management of patients [19, 21, 35]. In this study, compared to PTCs without any mutation, PTCs with the TERT+BRAF mutation were associated with non-parallel orientation, and PTCs with the BRAF mutation alone were related to incidences of non-parallel orientation and hypoechogenicity based on US. There was a strong relationship between the number of genetic mutations and the proportions of malignant US features including hypoechogenicity, non-parallel orientation, spiculated/microlobulated margin, and microcalcifications. As the number of genetic mutations increased, the probability of having malignant US features increased. Therefore, US features of PTCs can serve to predict the presence of genetic mutations.

Recently, Liu et al. reported the value of preoperatively testing for TERT mutations in thyroid fine needle aspiration (FNA) specimens [36]. In their study, they found TERT promoter mutations in 0% (0/179) of benign thyroid nodules and 7.0% (9/129) of differentiated thyroid cancers, representing 100% diagnostic specificity. Therefore, we reasoned that the combination of FNA with TERT promoter mutation analysis might be helpful to select the appropriate management and to predict clinical prognosis, particularly when thyroid nodules show non-parallel orientation by US.

Our study has several limitations. First, this was a retrospective study because we analyzed the presence of BRAF and TERT promoter mutations in the specimens that had been resected at the time of surgery. We excluded 2255 PTC cases according to the exclusion criteria prior to the mutational analysis. Therefore, there was the possibility of selection bias associated with this data collection method. We are not sure that 150 patients out of 2425 (6.2%) can represent general PTC patients. However, current international guidelines lead to a trend toward more conservative approaches to the diagnosis and treatment of this disease [30, 37]. Small tumors less than 1 cm are no longer a concern in the present setting. Therefore, current study subjects are more appropriate to guide the management of this disease. Further prospective studies with larger sample sizes and long-term follow up periods are recommended to validate the study results. Second, our overall sample size was relatively small. The incidence of PTC with TERT mutation alone was very rare (1/151, 0.7%) in our original data and we did not include it for the statistical analysis. Third, in this study, the mean size of PTCs was 3.0 cm (range, 0.5–8.0 cm), which was relatively large. However, there were no differences in tumor size among the three groups.

In conclusion, PTCs with no mutation, with BRAF mutation alone, and with both TERT promoter and BRAF mutations linearly increase in the probability of displaying malignant US features. In PTCs, the coexistence of BRAF mutation with TERT promoter mutation is more strongly correlated with recurrence and mortality than BRAF mutation alone. Therefore, the TERT promoter mutation might be a useful prognostic molecular marker for risk stratification and further management in patients with PTC, and PTC with both TERT promoter and BRAF mutations can be suspected based on the US features.

## MATERIALS AND METHODS

### Population

The Institutional Review Board at Samsung Medical Center, Seoul, Korea, approved this retrospective study and the use of human thyroid tissue. Informed patient consent was waived. Between October 1994 and December 2004, 2425 consecutive patients were diagnosed as PTC at our institution. We excluded 2255 patients with the following conditions: tumors less than 0.5 cm in size including incidental PTCs by operation for benign lesions (n = 993), cases that was unsuitable for sampling retrieval of tumors less than 1 cm (n = 711), lack of preoperative US at our institution (n = 279), and clinical or imaging follow up duration less than 60 months (n = 272). Then, genomic DNA sequencing was performed to identify BRAF and TERT promoter mutations. We included only one sample per patient in all cases. We chose the largest index tumor in case of multifocal tumor. In total, 170 PTC samples were screened for mutational analysis. DNA sequencing was successful in 163 samples (95.9%) for BRAF mutations and 151 samples (88.8%) for TERT promoter mutations. We excluded one case with TERT promoter mutation alone to achieve statistical analysis. Finally, the study included 150 PTCs in 150 patients (26 males and 124 females; age range, 15.8–81.4 years; mean age, 43.7 years) who treated by total thyroidectomy and neck dissection (104 central and 46 central/lateral neck dissections) and who had the successful results of DNA sequencing for BRAF and TERT promoter mutations. The mean tumor size was 3.0 cm, ranging from 0.5 to 8.0 cm. After surgery, all patients received thyrotropin suppressive therapy, and most of the patients (n = 146, 97.3%) underwent radioiodine scan with therapeutic (n = 142) or diagnostic (n = 4) dose. At the study period, treatment protocol for PTCs larger than 1cm was total thyroidectomy and postsurgical radioiodine administration for most of cases [38–40]. Subtotal thyroidectomy depended on the preference of the surgeon to reduce the complications. A therapeutic neck dissection was performed when nodal metastasis was apparent clinically (preoperatively or intraoperatively) or by imaging, especially for nodes of lateral compartment. A prophylactic central compartment dissection was carried out although nodal metastasis was not detected clinically or by imaging.

Routine follow up of postoperative status included serum thyroglobulin (Tg) with anti-Tg antibody, thyroid function test, neck US, and chest X-ray. Follow up postoperative US examination was routinely performed for all patients to evaluate the thyroid bed and cervical nodal compartments at 6 months and then annually for at least 5 years. Some patients underwent neck computed tomography (CT) scan (n = 11), positron emission tomography-CT scan (n = 2), or both (n = 2) to evaluate the recurrence during the follow up period. We obtained thyroid tumor specimens for genetic analysis and retrospectively collected medical records, vital status, and death certificates. All tumor factors including tumor size, multiplicity, extrathyroidal extension, surgical margin involvement, lymph node metastasis, and aggressive subtype were evaluated based on the pathology results after surgery. Pathologic stages of PTC were defined based on the American Joint Committee on Cancer staging system.

### Mutational analysis

All mutational analyses were performed after surgical and radioiodine treatments of patients; therefore, the genetic results had no influence on the treatment decisions. DNA samples for molecular analysis were extracted from postoperative surgical specimens using a Qiagen DNA FFPE Tissue Kit (Qiagen, Hilden, Germany) according to the manufacturer’s instructions. For BRAF T1799A, direct sequencing after conventional polymerase chain reaction (PCR) and mutant enrichment with 3′-modified oligonucleotides-PCR (MEMO-PCR) were performed. DNA sequences from both methods were compared with the normal BRAF gene sequence, specifically exon 15, from the GenBank Database (GenBank accession number NM 004333.4) using sequence assembly software (Gene Codes Corp., Ann Arbor, MI, USA). For TERT C228T, semi-nested PCR was performed to identify TERT promoter mutations. PCR reactions were performed using a GeneAmp PCR system 9700 thermal cycler (Applied Biosystems, Foster City, CA). Cycle sequencing was performed using Big Dye Terminator Cycle Sequencing Ready Reaction kits on an ABI 3730xl Genetic Analyzer (Applied Biosystems). According to the existence of BRAF T1799A or TERT C228T mutations, we categorized patients into three groups as the no mutation group, the BRAF mutation alone group, and the coexistence group of TERT promoter and BRAF mutations (i.e. TERT+BRAF mutation group).

### US imaging analysis

The Logiq 700 scanner (General Electric Healthcare, Milwaukee, WI), an HDI 5000 scanner (Philips Ultrasound, Bothell, WA), or the IU22 scanner (Philips Medical Systems, Bothell, WA) with a 5- to 12-MHz linear-array transducer were used for thyroid US examinations. Four radiologists specialized in thyroid imaging, with 5–10 years of experience and four senior residents, performed preoperative US examinations during the study period. All US images were retrospectively reviewed and interpreted with consensus by two faculty radiologists (S.Y.H. and J.H.S.) who were blinded to the clinicopathological features and mutational analysis results.

According to the K-TIRADS [30], all thyroid nodules were described for composition, internal echogenicity, orientation, margin, calcifications, and final K-TIRADS category. The internal composition was categorized as solid (no obvious cystic content), predominantly solid (≤50% of the cystic portion), predominantly cystic (> 50% of the cystic portion), or cystic (no obvious solid content). The echogenicity was categorized as marked hypoechoic, hypoechoic, and hyper- or isoechoic. The orientation was classified as parallel or non-parallel. The margin was classified as being circumscribed, spiculated/microlobulated, or ill-defined. If calcifications were present, they were classified as microcalcifications (≤1 mm in size), macrocalcifications (>1 mm in size with posterior shadowing), or rim calcification. US findings of hypoechogenicity, non-parallel orientation, spiculated/microlobulated margin, and microcalcifications are considered indicative of malignancy. The final K-TIRADS assessments of the thyroid nodules were classified into 5 groups: category 1, no nodule; category 2, benign nodule (spongiform, pure cyst, or partially cystic nodule with comet tail artifact); category 3, low suspicion nodule (partially cystic or isohyperechoic nodule without any of the three suspicious US features including microcalcification, nonparallel orientation, or spiculated/microlobulated margin); category 4, intermediate suspicion nodule (solid hypoechoic nodule without any of the three suspicious US features or partially cystic or isohyperechoic nodule with any of the three suspicious US features); category 5, high suspicion nodule (solid hypoechoic nodule with any of the three suspicious US features).

### Statistical analysis

Clinicopathological features including US features of the three groups were compared using the χ^2^-test or Fisher’s exact test for categorical variables and the ANOVA test or the Kruskal-Wallis test for continuous variables. Post-hoc pairwise analysis was performed using a Bonferroni correction. Because Bonferroni correction adapts the threshold for significance for multiple comparisons by dividing the significance level by the number of tests performed, a *P* value of .0167 (.05/3) was considered significant in this study. The Cochran-Armitage trend test was performed to detect a trend in the relationship between the presence of genetic mutations and the suspicious malignant features of thyroid nodules on US. A *P* value for the trend calculated by Cochran-Armitage trend test is considered significant if *P* value <.1.

Multinomial logistic regression analysis was used to assess independent associations between mutational analysis results and clinicopathological features including US features. No mutation and BRAF mutation alone groups served as references. The results are presented as odds ratios with 95% confidence intervals.

The recurrence-free interval was calculated from the date of surgery to the first date of recurrence or distant metastasis. Patients without any of these events were censored at the time of death from any cause or at last follow-up. Overall survival was calculated as the interval from surgery to death from any cause, censoring for living patients at the most recent contact date. The rate of recurrence or death according to mutational analysis results was estimated by the Kaplan-Meier method. The univariate influence of prognostic factors on study end-points was analyzed using the log-rank test.

Statistical significance was accepted with a two-sided *P* value < .05. All statistical analyses were executed using SAS version 9.4 (SAS Institute, Cary, NC) and R 3.2.2 (Vienna, Austria; http://www.R-project.org/).
